# Effects of Total Flavonoids From *Rubus chingii* Hu on the Hypothalamic–Pituitary–Ovarian Axis and Inflammatory Response in Polycystic Ovary Syndrome Rats

**DOI:** 10.1002/fsn3.72004

**Published:** 2026-06-07

**Authors:** Mengfan Peng, Baosong Liu, Lanyue Cao, Chuan Rao, Yingying Sun, Caixia Li, Hao Chen, Lei Yang

**Affiliations:** ^1^ Faculty of Medicine, HuangHuai University Zhumadian Henan China; ^2^ Department of Pharmacy Zhumadian Traditional Chinese Medicine Hospital Zhumadian Henan China; ^3^ Key Laboratory of Cardiovascular and Cerebrovascular Diseases, Tianfang Pharmaceutical Co., Ltd. Zhumadian Henan China; ^4^ Department of Clinical Pharmacy The First Affiliated Hospital of Xinxiang Medical University Xinxiang Henan China; ^5^ Department of Scientific Research Section The First People's Hospital of Zhumadian, Afliated Hospital of Huanghuai University Zhumadian Henan China

**Keywords:** hypothalamic–pituitary–ovarian axis, inflammatory response, NLRP3 inflammasome, polycystic ovary syndrome, Rubus chingii Hu total flavonoids

## Abstract

Polycystic ovary syndrome (PCOS) is a common reproductive disorder in women of childbearing age, posing serious risks to both physical and mental health. Dysregulation of the hypothalamic–pituitary–ovarian (HPO) axis and inflammatory responses are key pathological mechanisms, yet there are currently no specific therapeutic drugs available. Therefore, this study investigates the ameliorative effects of total flavonoids from *Rubus chingii* Hu (RHF) on HPO axis function and inflammatory response in a rat model of PCOS induced by letrozole. The results demonstrated that RHF restored the disrupted estrous cycle in PCOS rats. It significantly reduced ovarian weight, index, length, width, area, and volume. Moreover, RHF treatment decreased the ovarian mRNA levels of BCL‐2‐associated X protein (Bax) and aspartate specific cysteine protease (Caspase)‐3 while increasing that of B‐cell lymphoma‐2 (Bcl‐2), collectively ameliorating ovarian pathological changes. Concurrently, RHF significantly lowered serum levels of gonadotropin‐releasing hormone (GnRH), luteinizing hormone (LH), and testosterone (T), while elevating follicle‐stimulating hormone (FSH), estradiol (E_2_), and sex hormone‐binding globulin (SHBG), indicating its potential to mitigate hypothalamic–pituitary–ovarian (HPO) axis dysfunction. Furthermore, RHF reduced peripheral blood counts of white blood cells (WBC), platelets (PLT), red blood cell distribution width (RDW), the neutrophil‐to‐lymphocyte (NLR), monocyte‐to‐lymphocyte (MLR), and platelet‐to‐lymphocyte (PLR) ratios, as well as serum interleukin‐18 (IL‐18), IL‐1β, tumor necrosis factor‐α (TNF‐α), and IL‐6. In ovarian tissue, RHF downregulated the protein and mRNA expression of NLRP3, caspase‐1, the N‐terminal fragment of gasdermin D (GSDMD‐NT), IL‐18, and IL‐1β, and lowered the mRNA levels of IL‐6 and TNF‐α, thereby attenuating both systemic and local ovarian inflammatory responses. Taken together, these findings demonstrate that RHF confers protective effects in PCOS rats, which are associated with the amelioration of HPO axis function and suppression of inflammatory responses.

## Graphical abstract

RHF improves sex hormone secretion imbalance, restores the estrous cycle, inhibits ovarian hyperplasia and pathology, and reduces systemic and local ovarian inflammation in PCOS rats via enhancing HPO axis function and suppressing NLRP3 inflammasome signaling pathway activation.

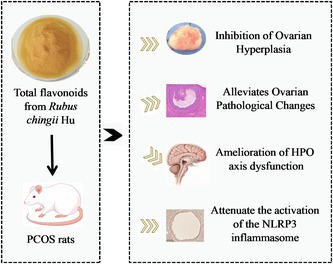

## 1 Introduction

Polycystic ovary syndrome (PCOS) is a multi‐faceted reproductive endocrine disorder that affects approximately 5%–10% of women worldwide. Its typical characteristics include irregular menstrual cycles, polycystic morphological changes in the ovaries, and clinical or biochemical hyperandrogenism (Simon et al. 2024). PCOS not only causes reproductive disorders in women but also increases the risk of developing conditions such as metabolic syndrome and cardiovascular and cerebrovascular diseases, posing a serious threat to women's physical and mental health (Su et al. 2025). Dysfunction of the HPO axis and the resulting imbalance in sex hormone secretion represent a crucial pathophysiological mechanism in PCOS. In particular, aberrant pulsatile secretion of GnRH leads to disrupted pituitary gonadotropin secretion, which serves as a key driver of abnormal ovarian secretion of E_2_ and T (Zuchelo et al. 2024). Studies have shown that elevated levels of inflammatory markers, such as IL‐18, IL‐1β, IL‐6, and TNF‐α are commonly observed in patients with PCOS. These markers not only impair ovarian function but are also closely associated with pathological processes including abnormal androgen levels, ovulatory dysfunction, and miscarriage (Deng et al. 2024), representing another key driver in the pathogenesis and progression of PCOS. Therefore, improving HPO axis function and controlling inflammatory response play crucial roles in the management of PCOS.

*Rubus chingii* Hu, first documented in the “Shen Nong's Materia Medica” is a commonly used traditional Chinese medicine known for its effects of tonifying the kidneys and consolidating essence. *Rubus chingii* Hu is rich in nutrients, possesses a sweet and sour taste, and has a high safety profile. It has been recommended by the Food and Agriculture Organization (FAO) as a third‐generation fruit and was listed as a food‐medicine resource in 2015 (He et al. 2023). Modern research has shown that *Rubus chingii* Hu contains chemical components such as flavonoids, terpenoids, alkaloids, and polysaccharides, and exhibits pharmacological effects including regulating the gonadal axis, anti‐inflammation, anti‐oxidation, anti‐aging, and enhancing immunity (Hen et al. 2025; Li et al. 2025). Studies have reported that the combination of *Rubus chingii* Hu with acupoint catgut embedding can regulate sex hormone levels, improve inflammatory status, and alleviate ovarian pathological changes in PCOS‐IR rats (Li 2021). However, the specific active components and underlying mechanisms of action remain unclear, which to some extent limits its broader application. It is noteworthy that among its numerous components, flavonoids have been identified as the primary compounds in *Rubus chingii* Hu and serve as the key material basis for its pharmacological effects. However, no experimental studies have yet investigated the intervention of total flavonoids from *Rubus chingii* Hu (RHF) on PCOS. Appropriate animal models are particularly important for pharmacological efficacy research. Among various methods for constructing PCOS animal models, letrozole, as an aromatase inhibitor, not only inhibits aromatase activity in ovarian tissue to cause locally elevated androgen levels but also affects the function of the HPO axis, suppresses the secretion of E_2_ in the body, and thereby creates an environment of hyperandrogenemia (Yang et al. 2018). Rats with letrozole‐induced PCOS also exhibit typical PCOS features such as disrupted estrous cycles, abnormal ovarian histology, inflammatory responses, and insulin resistance (Wu et al. 2022; Danduga et al. 2024), which encompass the two core pathological characteristics of our study: HPO axis dysfunction and inflammatory responses. Therefore, this study focuses on RHF as the research subject, with the HPO axis and the NLRP3 inflammasome signaling pathway as the main investigation targets, to examine the regulatory effects of RHF on PCOS rats and explore its potential mechanisms of action.

## 2 Materials and Methods

### 2.1 Reagents and Kits

**FIGURE 1:**
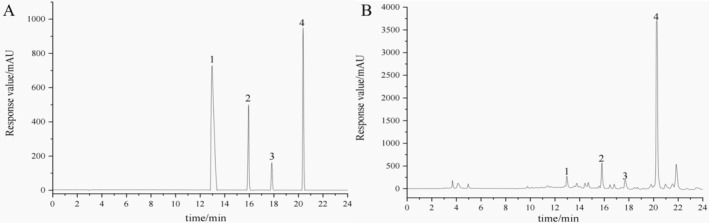
HPLC chromatogram of RHF. (A) Reference standard. (B) Sample of RHF. 1 rutin; 2 isoquercitrin; 3 astragalin; 4 kaempferol‐3‐*O*‐rutinoside.

RHF the main extraction and purification procedures are as follows: crush the *Rubus chingii* Hu, add 15 times the amount of 65% ethanol, soak for 4 h, followed by ultrasonic extraction twice with an ultrasonic temperature of 60°C, ultrasonic power of 300 W, for 1 h each time. After extraction, combine the two extracts and concentrate to 0.3 g/mL. Purification was carried out using D101 macroporous resin with an aspect ratio of 1:6. The sample was loaded at 2 BV, washed with 2 BV of water, and then eluted with 4 BV of 60% ethanol. The 60% ethanol eluate was collected, concentrated under reduced pressure with reflux, and dried to constant weight to obtain the RHF, which was then sealed and stored in a desiccator. Finally, using rutin as the standard, the total flavonoid content in *Rubus chingii* Hu was determined by UV spectrophotometry to be 58.37%. The main components of the total flavonoids from raspberry were analyzed using high‐performance liquid chromatography (HPLC), which revealed the presence of kaempferol‐3‐*O*‐rutinoside, rutin, isoquercitrin, and astragalin, as shown in Figure 1. The gradient elution program for HPLC is detailed in Table 1. Letrozole tablets were purchased from Zhejiang Hisun Pharmaceutical Co. Ltd. (Zhejiang, China). Dc‐35 was purchased from Bayer Healthcare Co. Ltd. Guangzhou Branch (Guangzhou, China). T and E_2_ enzyme‐linked immunosorbent assay (ELISA) kits were purchased from Jiangsu Meimian lndustrial Co. Ltd. (Jiangsu, China). GnRH, LH, FSH, and SHBG ELISA kits were purchased from Suzhou Calvin Biotechnology Co. Ltd. (Suzhou, China). Rabbit Anti‐NLRP3 was purchased from Bioss (batch no. bs‐6655R). Rabbit Anti‐Caspase‐1 was purchased from Proteintech (batch no. 22915‐1‐AP). Rabbit Anti‐GSDMD‐NT (batch no. U03075411) and Rabbit Anti‐IL‐18 (batch no. U02061127) were purchased from Myriads biotechnology Co. Ltd. Rabbit Anti‐IL‐1β was purchased from Servebio (batch no. GB11113).

**TABLE 1:** HPLC gradient elution program.

Time/min	Acetonitrile (%)	0.1% phosphoric acid in water (%)
0	5	95
3	5	95
10	40	60
20	70	30
30	90	10

### 2.2 Animals

42 healthy 6 weeks old SPF‐grade female SD rats were provided by Shandong Jinan Pengyue Laboratory Animal Breeding Co. Ltd., animal qualification certificate No. 370726241101650545. All the rats were housed in barrier systems under controlled environmental conditions and maintained at a controlled room temperature of 24°C–26°C, with a 12‐h light–dark cycle. All procedures involving rats were approved by the Ethics Committee of Hhuanghuai University.

### 2.3 PCOS Induction and Grouping (Zhang, Jiang, et al. 2025; Magdy et al. 2024)

Following a 3‐day adaptive feeding period, 8 rats were randomly selected using a random number table to serve as the control group. The remaining 34 rats were administered letrozole (1 mg/kg·day) via intragastric gavage once daily for 21 days to establish the PCOS model. The control group received an equivalent volume of the solvent. From day 14 to 21 of modeling, vaginal smears were performed to select successfully modeled PCOS rats, which were randomly divided into the model group, Dc‐35 group (0.3392 mg/kg·day), high‐dose RHF group (RHF‐H, 200 mg/kg·day), and low‐dose RHF group (RHF‐L, 100 mg/kg·day), with 8 rats in each group. All groups received continuous administration for 21 days, while the control group and the model group were given an equivalent volume of solvent. The grouping procedure was performed by personnel who were not involved in the subsequent interventions or outcome assessments. Blinding was implemented during data collection and analysis. For instance, in histopathological and immunohistochemical evaluations, all samples were re‐coded with random numbers by one investigator to conceal group identity. Subsequently, another investigator, who was blinded to the group allocation, performed the microscopic examination, image acquisition, and semi‐quantitative analysis. The group codes were only revealed after all analyses were completed for statistical processing.

### 2.4 Estrous Cycle Monitoring (Dou et al. 2024)

From day 12 to day 21 of drug intervention, vaginal smear cytology was performed to monitor the estrous cycle in each group of rats. All vaginal smear observations and estrous cycle stage determinations were performed by an investigator who was blinded to the experimental group allocations. This ensured that the analysis was free from observer bias. Regarding the estrous cycle criteria, the estrous cycle stages (proestrus, estrus, metestrus, diestrus) were strictly determined according to methods described in the literature (Dong et al. 2025; Hu et al. 2024), which guaranteed the objectivity and consistency of the scoring.

### 2.5 Analysis of Ovarian Weight, Index, Long Diameters, Short Diameters, Area, and Volume (Peng et al. 2021)

Ovarian tissues were harvested and weighed to calculate the organ index (ovarian index = ovarian weight (mg)/body weight (g)). Following weighing, the long and short diameters (L and S) of the ovaries were measured using a vernier caliper. The ovarian area (MOA = L × S) and ovarian volume (V = 4.19 × [(L + S)/2]^3^) [12] were subsequently calculated.

### 2.6 Determination of Peripheral Blood Cells and Serum Hormones in Rats

At the end of the experiment, blood was collected from the abdominal aorta. One portion was collected in anticoagulant tubes containing EDTAK2. Following the operating procedures of the hematology analyzer, the WBC count, PLT count, and RDW in the peripheral blood were measured. The MLR, PLR, and NLR were calculated to evaluate the inflammatory response.

The other portion was collected in plain tubes, left to stand, and then centrifuged to obtain serum, which was used to measure the levels of GnRH, LH, FSH, E_2_, T, and SHBG, in order to assess the impact on hormone secretion. Among them, GnRH, LH, FSH, SHBG, IL‐18, IL‐1β, IL‐6, and TNF‐α ELISA kits were purchased from Suzhou Calvin Biotechnology Co. Ltd., while E_2_ and T ELISA kits were purchased from Jiangsu meimian industrial Co. Ltd. Detection procedure and quality control: All operations were strictly performed in accordance with the kit instructions. Measurement for each indicator was completed on the same 96‐well plate, with standard curves and blank wells included to minimize inter‐plate variation. Detection range and sensitivity: The standard detection ranges for each indicator are as follows: GnRH 0–80 mIU/mL, LH 0–48 mIU/mL, FSH 0–12 IU/mL, IL‐18 0–120 pg/mL, IL‐1β 0–40 pg/mL, IL‐6 0–160 pg/mL, TNF‐α 0–320 pg/mL, E_2_ 5–80 ng/mL, T 0.5–8 nmol/L, SHBG 0–120 nmol/L. The detection sensitivities provided in the ELISA kit manuals were all lower than the minimum values detected in the samples. Standard Curve and Linearity: The correlation coefficients (R^2^) of all standard curves were greater than 0.99 (GnRH 0.9912, LH 0.9995, FSH 0.9949, IL‐18 0.9967, IL‐1β 0.9984, IL‐6 0.9984, TNF‐α 0.9949, E_2_ 0.9939, T 0.9939, SHBG 0.9983), indicating excellent fit. With the exception of three T samples (see below), all sample measurements fell within the linear range of their respective standard curves.

### 2.7 Histopathological Analysis of Ovary in Rats

After blood collection, the rats were euthanized by cervical dislocation, and the ovarian tissues were harvested. The tissues were fixed in 4% paraformaldehyde solution, followed by gradient dehydration and embedding in paraffin blocks. For each ovarian sample, after paraffin embedding, serial sections were made along its maximum equatorial plane. The most representative 4 μm thick section was selected for hematoxylin–eosin (HE) staining. Two researchers, blinded to the experimental groups, counted the corpora lutea, atretic follicles, and cystic follicles in the entire section under a light microscope. The final data represent the average of the counts from the two observers. The classification criteria for corpus luteum, cystic follicles, and atretic follicles are as follows:

Corpus luteum: The main characteristics are clusters of large, pale‐staining luteal cells with abundant cytoplasm, often surrounding a central cavity containing fibrin remnants or lacking a distinct lumen. This structure is highly vascularized.

Cystic follicle: Characterized by a follicle diameter larger than that of a normal follicle. The cyst wall is thin in cross‐section, composed of several layers of granulosa and theca cells. The inner surface of the cavity is smooth, containing clear fluid, and the oocyte is absent.

Atretic follicle: The primary features include chromatin condensation, wrinkling of the nuclear membrane, pyknosis of granulosa cells, detachment of granulosa cells from the granulosa layer suspending in the follicular fluid, disintegration of the cumulus oophorus, abnormal division or fragmentation of the oocyte, vitrification and thickening of the zona pellucida, and cytoplasmic fragmentation, among other changes.

### 2.8 Immunohistochemical (IHC) Staining

Antigens were retrieved by autoclaving the tissues at 120°C for 15 min in citrate buffer (pH 6.0) and incubating them in 3% (v/v) hydrogen peroxide for 8 min to inactivate endogenous catalase. The sections were then blocked with 10% (v/v) horse serum. Then, the ovary sections were incubated with anti‐NLRP3 (1:300), anti‐Caspase‐1 (1:200), anti‐GSDMD‐NT (1:200), anti‐IL‐18 (1:300), and anti‐IL‐1β (1:200) antibodies at 4°C overnight, with an HRP‐conjugated secondary antibody for 1 h, and with streptavidin HRP. Semi‐quantitative analysis of the results for NLRP3, Caspase‐1, GSDMD‐NT, IL‐18, and IL‐1β was performed using ImageJ. The methods for selecting regions of interest (ROI), background correction, and staining quantification are described as follows. ROI Selection: For each ovarian tissue section, five non‐overlapping, representative fields of view (e.g., areas containing ovarian follicles and stroma) were selected at 200× magnification for analysis. Areas with artifacts, folds, or excessive bleeding were systematically excluded. Background Correction: For each image, a background region with no specific staining was selected. The average optical density of this background region was measured and subtracted from the optical density values measured within the specific ROIs to correct for variations in background illumination and non‐specific staining. Staining Quantification: The “Color Deconvolution” plugin was used to separate the brown DAB signal from the blue hematoxylin counterstain. The threshold for positive DAB staining was set consistently across all images based on control slides (negative and positive controls, as described in the methods). The integrated optical density of the DAB‐positive area within each ROI was then measured. The final value for each sample was expressed as the average integrated optical density per unit area (AOD) across the analyzed fields.

### 2.9 Quantitative Real‐Time PCR

First, total RNA was extracted from ovarian tissues using the FastPure Cell/Tissue Total RNA Isolation Kit V2 (Vazyme, RC112‐01). Subsequently, 1 μg of total RNA was reverse transcribed into cDNA following the instructions of the HyperScript RT SuperMix for qPCR kit (Vazyme, R323). Finally, real‐time PCR was performed using a HyperScript 2 × SYBR Green qPCR Master Mix (Servicebio INC, G3326‐05) according to the manufacturer's instructions on an ABI system (Applied Biosystems, Thermo Fisher Scientific Inc., MA, USA). The relative expression was calculated using the 2^–ΔΔCt^ method. The following oligonucleotides were used as PCR primers: *NLRP3* forward: 5′‐TAAGAAGGACCAGCCAGAGTG‐3′; *NLRP3* reverse: 5′‐CTGGGTGTAGCGTCTGTTGAG‐3′; *Caspase‐1* forward: 5′‐TGCCTGGTCTT GTGACTTGGAG‐3′; *Caspase‐1* reverse: 5′‐TGTCCTGGGAAGAGGTAGAAACG‐3′; *GSDMD* forward: 5′‐GGAGATCATGCAACGTGAAAGG‐3′; *GSDMD* reverse: 5′‐TCTTCTTCCGGCTTTGGTGG‐3′; *IL‐18* forward: 5′‐GGAATCAGACCAC TTTGGCAGA‐3′; *IL‐18* reverse: 5′‐GTCTGGTCTGGGATTCGTTGG‐3′; *IL‐1β* forward: 5′‐GAACAACAAAAATGCCTCGTGC‐3′; *IL‐1β* reverse: 5′‐GACAAA CCGCTTTTCCATCTTCT‐3′; *TNF‐α* forward: 5′‐CCACCACGCTCTTCTGTCTAC TG‐3′; *TNF‐α* reverse: 5′‐TGGGCTACGGGCTTGTCACT‐3′; *IL‐6* forward: 5′‐AAGAGACTTCCAGCCAGTTGCC‐3′; *IL‐6* reverse: 5′‐TGTGGGTGGTATCCTC TGTGAAG‐3′; *BAX* forward: 5′‐TTTGCTACAGGGTTTCATCCAG‐3′; *BAX* reverse: 5′‐GTTGTTGTCCAGTTCATCGCC‐3′; *Bcl‐2* forward: 5′‐TTGTGGCCTTCTTTGA GTTCG‐3′; *Bcl‐2* reverse: 5′‐GCATCCCAGCCTCCGTTAT‐3′; *β‐actin* forward: 5′‐TGCTATGTTGCCCTAGACTTCG‐3′; *β‐actin* reverse: 5′‐GTTGGCATAGAGGT CTTTACGG‐3′.

### 2.10 Statistical Analysis

The date were expressed as the mean ± standard deviation (SD) and analyzed with SPSS v. 21.0 for Windows (IBM Corp., Armonk, NY, USA). Group comparisons were conducted with a homogeneity of variance test followed by one‐way ANOVA. The Games‐Howell method was used when the variances were uneven, and the Bonferroni post‐test was used for comparison between two groups. *p* < 0.05 was considered statistically significant.

## 3 Results

### 3.1 Effects of RHF on the Estrous Cycle in PCOS Rats

**FIGURE 2:**
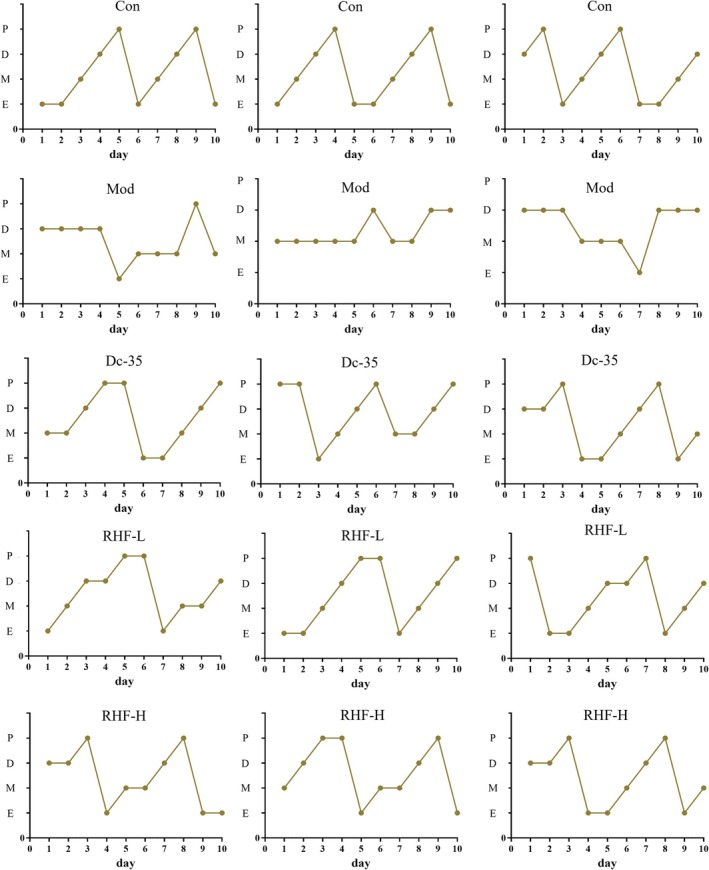
Estrous cycles of the rats in the 5 groups.

Estrous cycle disruption is a hallmark characteristic of animal models of PCOS. It serves as a useful indicator for both evaluating the success of the model and assessing drug efficacy. In our experiments, we observed that the estrous cycle in the Con group rats lasted approximately 4–5 days, with the four stages occurring in a regular sequence. The proestrus stage was characterized primarily by nucleated epithelial cells; the estrus stage was dominated by cornified squamous epithelial cells; the metestrus stage featured a mixture of leukocytes, cornified squamous epithelial cells, and nucleated epithelial cells; and the diestrus stage was primarily comprised of leukocytes. In the Mon group, the rats exhibited disrupted estrous cycles, predominantly remaining in the metestrus stage. After two estrous cycles, the estrous stages recurred regularly in the Dc‐35, RHF‐L, and RHF‐H groups (Figure 2), demonstrating that RHF can improve ovarian function and restore the estrous cycle in PCOS rats.

### 3.2 Effects of RHF on the Ovarian Weight, Index, Long Diameters, Short Diameters, Area, and Volume in PCOS Rats

**FIGURE 3:**
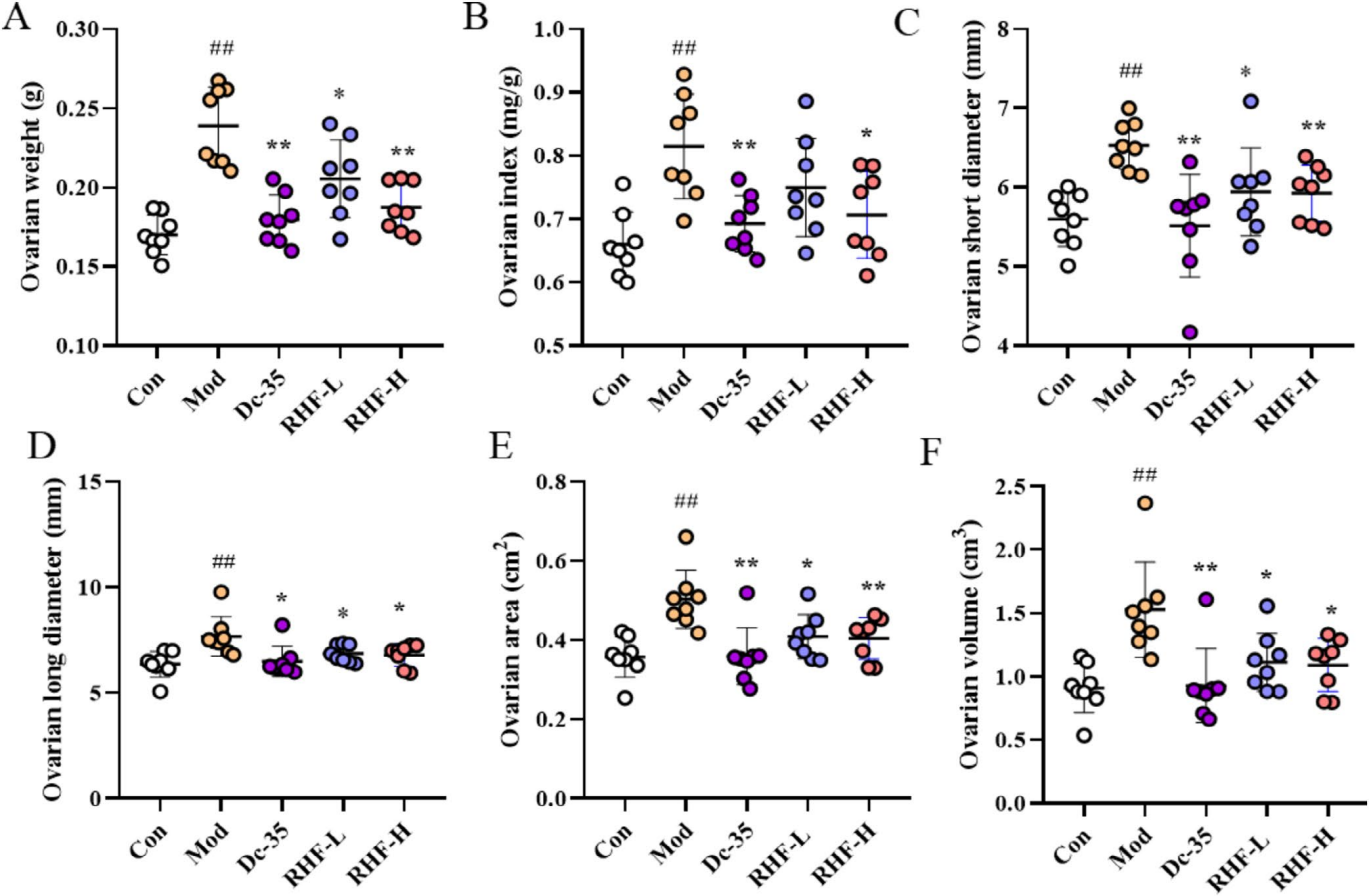
Effects of RHF on the ovarian weight, index, long diameters, short diameters, area, and volume in PCOS rats. (A) Ovarian weight. (B) Ovarian index. (C) Ovarian short diameters. (D) Ovarian long diameters. (E) Ovarian area. (F) Ovarian volume. Data were shown as mean ± SD (*n* = 8) ^##^ *p* < 0.01 Con group versus the Mod group. *p < 0.05 versus the Mod group. **p < 0.01 versus the Mod group.

Compared to the Con group, the ovarian weight and ovarian index were significantly increased in the Mod group (*p* < 0.01). Compared to the Mod group, the Dc‐35 group showed a significant reduction in both ovarian weight and ovarian index (*p* < 0.01). The RHF‐L group exhibited a obviously decrease in ovarian weight (*p* < 0.05), while the ovarian index showed a decreasing trend (*p* > 0.05). The RHF‐H group demonstrated a significant decrease in ovarian weight (*p* < 0.01) and a obvious reduction in ovarian index (*p* < 0.05) (Figure 3A,B).

We subsequently measured the ovarian long and short diameters to assess potential differences in ovarian size. The results demonstrated that compared to the Con group, the long and short diameters of the ovaries were significantly increased in the Mod group (*p* < 0.01). Compared to the Mod group, both the Dc‐35 and RHF‐H groups showed a significant reduction in the short diameter (*p* < 0.01) and a obvious decrease in the long diameter (*p* < 0.05). The RHF‐L group exhibited a obvious decrease in both long and short diameters (*p* < 0.05) (Figure 3C,D).

Based on the observed changes in the long and short diameters of the ovaries in PCOS rats, we further evaluated the alterations in ovarian area and volume. The results indicated that compared to the Con group, both the ovarian area and volume were significantly increased in the Mod group (*p* < 0.01). Compared to the Mod group, the Dc‐35 group showed a significant reduction in both ovarian area and volume (*p* < 0.01). The RHF‐L group exhibited a obvious decrease in both ovarian area and volume (*p* < 0.05). The RHF‐H group demonstrated a significant decrease in ovarian area (*p* < 0.01) and a obvious reduction in ovarian volume (*p* < 0.05) (Figure 3E,F). These results suggest that pathological changes occur in the ovarian tissue of PCOS rats, manifesting as enlargement, while RHF can inhibit this ovarian enlargement and alleviate ovarian pathology.

### 3.3 Effects of RHF on the Pathological Changes in the Ovaries of PCOS Rats

**FIGURE 4:**
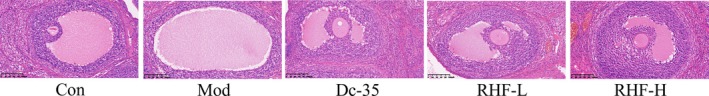
Effects of RHF on the pathological changes in the ovaries of PCOS rats (100×).

Based on the observed macroscopic pathological changes in the ovaries of PCOS rats (including alterations in weight, index, diameter, area, and volume), we further examined the histopathological changes using HE staining. Figure 4 illustrates that the Con group exhibited normal ovarian structure, with follicles at various developmental stages and corpora lutea scattered throughout the stroma. Theca cells were spindle‐shaped and arranged in an orderly fashion, while granulosa cells formed multiple, well‐organized layers. In contrast, the Mod group displayed significant pathological changes: a marked thickening of the theca cell layer, a reduction in the number of granulosa cell layers and mature follicles, a frequent absence of corona radiata or oocytes within follicles, a significant increase in atretic follicles and cystically dilated follicles, and a decreased distribution of corpora lutea. In the Dc‐35 and RHF‐H groups, follicular development at various stages was largely normal. The theca cell layer was slightly thickened, the number of granulosa cell layers was increased and neatly arranged, and corpora lutea and corpora albicantia were observed, with occasional cystic and atretic follicles present. The RHF‐L group showed a generally normal proportion of follicles at different stages, a relative reduction in the number of granulosa cell layers, visible corpora lutea, and fewer cystic and atretic follicles (Figure 4).

Statistical analysis revealed that compared to the Con group, the Mod group exhibited a significant decrease in the number of corpora lutea and a significant increase in the number of atretic follicles and cystic follicles within the ovarian tissue (*p* < 0.01). Compared to the Mod group, the Dc‐35 group showed a significant increase in the number of corpora lutea and a significant reduction in cystic follicles (*p* < 0.01), along with a obvious decrease in atretic follicles (*p* < 0.05). Both the RHF‐H and RHF‐L groups demonstrated a significant increase in the number of corpora lutea and a significant reduction in both atretic follicles and cystic follicles (*p* < 0.01) (Table 2).

**TABLE 2:** Effects of RHF on the counts of corpora lutea, cystic follicles, and atretic follicles in the ovaries of PCOS rats (*ō* ± 5, *n* = 5).

Group	Corpora lutea	Cystic follicles	Atretic follicles
Con	11.00 ± 2.24	0.80 ± 0.84	1.60 ± 1.14
Mod	4.20 ± 0.84^##^	6.00 ± 1.22^##^	6.20 ± 1.79^##^
Dc‐35	10.20 ± 1.48**	2.00 ± 1.58**	3.00 ± 1.41*
RHF‐L	8.40 ± 1.14**	1.40 ± 1.14**	2.40 ± 0.55**
RHF‐H	8.40 ± 1.52**	1.20 ± 0.84**	2.20 ± 1.10**

*Note:* ## *p* < 0.01 Con group vs. the Mod group. * *p* < 0.05 vs. the Mod group. ** *p* < 0.01 vs. the Mod group.

**FIGURE 5:**
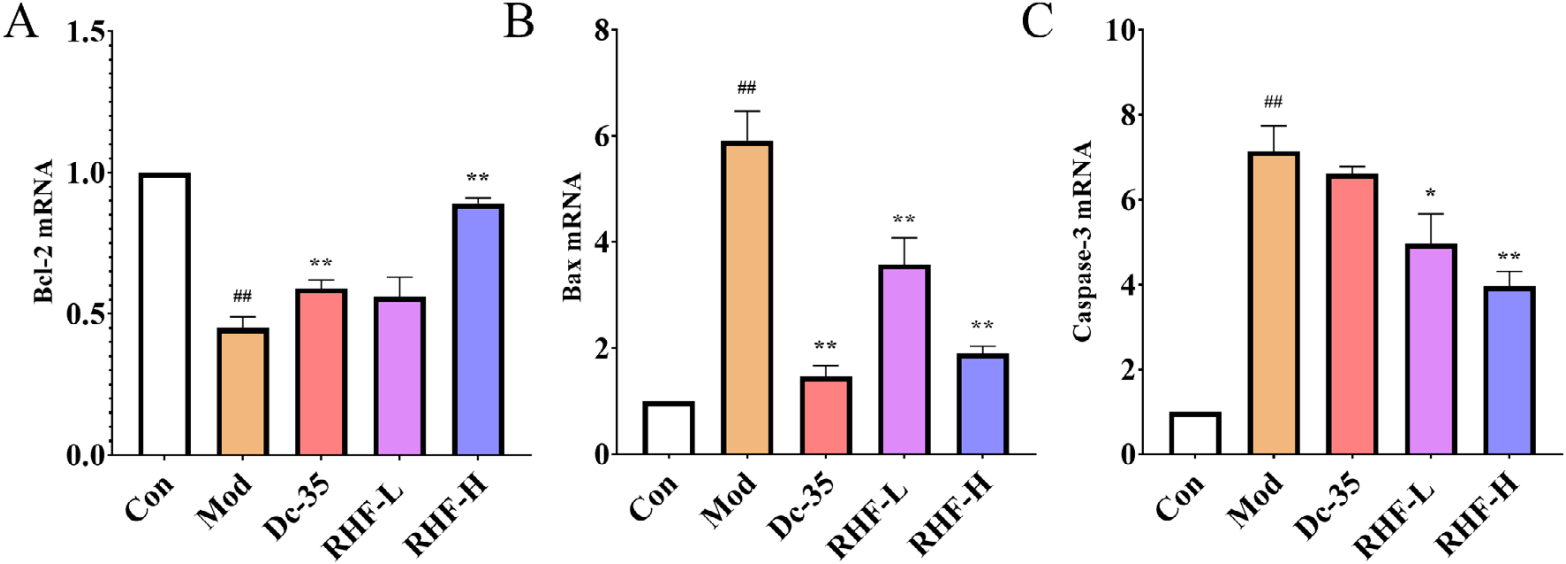
Effects of RHF on the expression of Bcl‐2, Bax, and Caspase‐1 in ovarian tissues of PCOS rats. (A) Bcl‐2 mRNA expression in the ovary. (B) Bax mRNA expression in the ovary. (C) Caspase‐1 mRNA expression in the ovary. Data were shown as mean ± SD (*n* = 3) ^##^ *p* < 0.01 Con group versus the Mod group. *p < 0.05 vs. the Mod group. **p < 0.01 versus the Mod group.

Apoptosis plays a crucial role in the development and maturation of follicles. Abnormal apoptosis is involved in the pathogenesis of PCOS, affecting ovarian function. Apoptosis is the programmed cell death within the organism, playing a significant role in maintaining the internal environment and the development of multiple systems. It is stringently regulated by several genes, including the Bcl‐2 family and the Caspase family (Zhang et al. 2023). Our results indicated that, compared with the Con group, the mRNA expression levels of Bax and Caspase‐3 in the ovarian tissues of rats in the Mod group were significantly increased, while the mRNA expression level of Bcl‐2 was significantly decreased (*p* < 0.01). Compared with the Mod group, the mRNA expression levels of Bax and Caspase‐3 in the ovarian tissues of rats in the RHF‐H group were significantly decreased, while the mRNA expression level of Bcl‐2 was significantly increased (*p* < 0.01). In the RHF‐L group, the mRNA expression level of Bax was significantly decreased (*p* < 0.01), the mRNA expression level of Caspase‐3 was markedly decreased (*p* < 0.05), and the mRNA expression level of Bcl‐2 showed an increasing trend (*p* > 0.05) (Figure 5).

### 3.4 Effects of RHF on Serum Hormone Levels (GnRH, LH, FSH, E_2_ , T, SHBG) in PCOS Rats

The ovary is the primary target organ for E_2_ and T secretion in females. Pathological changes in the ovaries suggest, to some extent, abnormal levels of E_2_ and T. Furthermore, aberrant E_2_ and T levels can exert negative feedback on the hypothalamus and pituitary gland tissue, leading to disruptions in the pulsatile release of GnRH from the hypothalamus and causing imbalances in the secretion of LH and FSH from the pituitary gland (Valera et al. 2025). Additionally, SHBG binds to sex hormones with a greater affinity for androgens than for estrogens. A decrease in its serum level is often associated with elevated androgen levels (Jafar et al. 2025). Consequently, we measured the serum levels of GnRH, LH, FSH, E_2_, T, and SHBG in rats from each group using ELISA kits.

**FIGURE 6:**
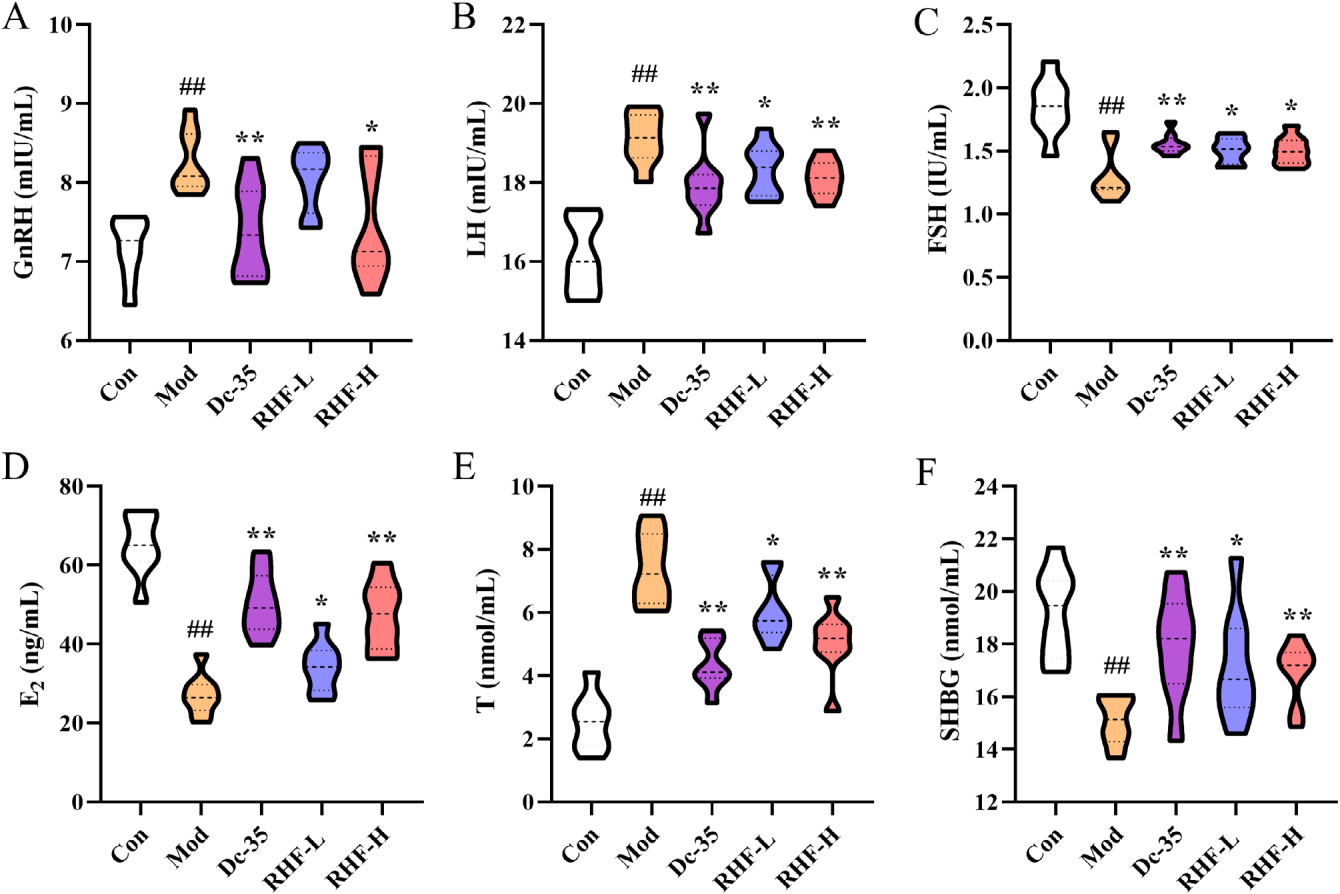
Effects of RHF on serum hormone levels (GnRH, LH, FSH, E_2_, T, SHBG) in PCOS rats. (A) GnRH level in serum. (B) LH level in serum. (C) FSH level in serum. (D) E_2_ level in serum. (E) T level in serum. (F) SHBG level in serum. Data were shown as mean ± SD (*n* = 8). ^##^ *p* < 0.01 Con group versus the Mod group. *p < 0.05 versus the Mod group. **p < 0.01 versus the Mod group.

The results demonstrated that compared to the Con group, the Mod group exhibited significantly increased serum levels of GnRH, LH, and T, along with significantly decreased levels of FSH, E_2_, and SHBG (*p* < 0.01). Compared to the Mod group, the Dc‐35 group showed significantly decreased serum levels of GnRH, LH, and T, and significantly increased levels of FSH, E_2_, and SHBG (*p* < 0.01). The RHF‐L group exhibited obviously decreased serum levels of LH and T, and obviously increased levels of FSH, E_2_, and SHBG (*p* < 0.05). In the RHF‐H group, serum GnRH levels were obviously decreased (*p* < 0.05), LH and T levels were markedly decreased (*p* < 0.01), FSH levels were obviously increased (*p* < 0.05), and E_2_ and SHBG levels were markedly increased (*p* < 0.01) (Figure 6).

### 3.5 Effect of RHF on Inflammatory Response in PCOS Rats

#### 3.5.1 Effects of RHF on the Blood Cell Count in PCOS Rats

Chronic low‐grade inflammation plays a critical role in the pathogenesis of PCOS, as it participates in the ovulatory process and influences hormone secretion. Complete blood count (CBC)‐related markers such as WBC count, PLT count, and RDW, along with their derived inflammatory indices—NLR, MLR, and PLR—can reflect the body's inflammatory status (Jia et al. 2025).

**FIGURE 7:**
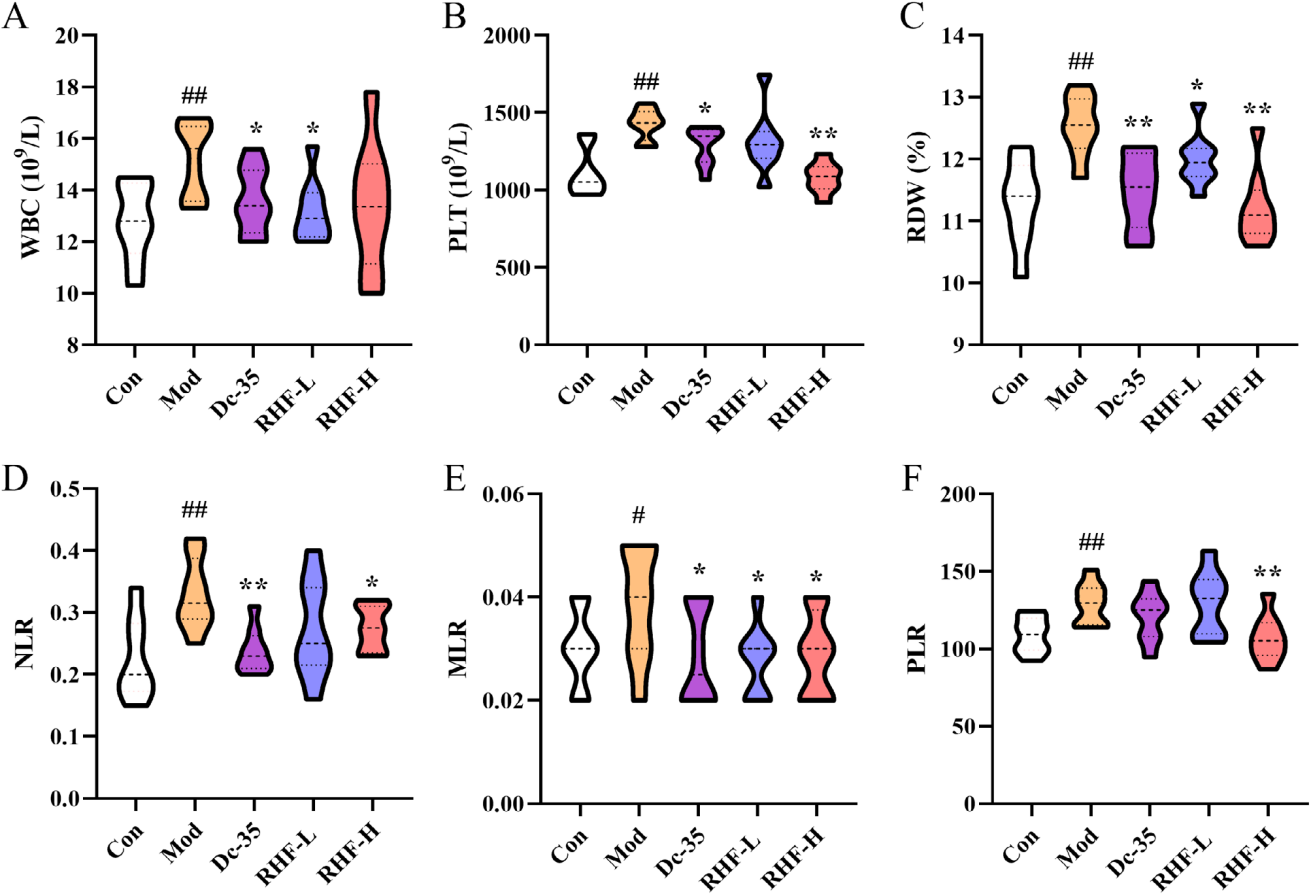
Effects of RHF on peripheral blood cell parameters in PCOS rats. (A) White blood cell count. (B) Platelet count. (C) Red cell distribution width coefficient of variation. (D) Neutrophil‐ to‐lymphocyte ratio. (E) Monocyte‐to‐lymphocyte ratio. (F) Platelet‐to‐lymphocyte ratio. Data were shown as mean ± SD (*n* = 8). ^##^ *p* < 0.01 Con group versus the Mod group. *p < 0.05 versus the Mod group. **p < 0.01 versus the Mod group.

Our study found that compared to the Con group, the Mod group exhibited significant increases in peripheral blood levels of WBC, PLT, RDW, NLR, MLR, and PLR (*p* < 0.01). Compared to the Mod group, the Dc‐35 group showed obviously reduced levels of WBC, PLT, and MLR (*p* < 0.05), and markedly decreased RDW and NLR (*p* < 0.01). In the RHF‐L group, WBC, RDW, and MLR were obviously lower (*p* < 0.05), while PLT, NLR, and PLR showed a decreasing trend (*p* > 0.05). The RHF‐H group demonstrated significantly lower PLT, RDW, and PLR (*p* < 0.01), as well as obviously reduced NLR and MLR (*p* < 0.05) (Figure 7). These results suggest that PCOS rats exhibit a systemic inflammatory response, and RHF can ameliorate this systemic inflammation in PCOS rats.

#### 3.5.2 Effects of RHF on the Ovarian NLRP3 Inflammasome Signaling Pathway in PCOS Rats

It has been reported that ovarian tissues of PCOS patients exhibit increased infiltration of lymphocytes and macrophages (Cao 2024), indicating the presence of not only systemic inflammation but also local ovarian inflammation in PCOS patients. In this pathological process of altered inflammatory microenvironment, the activation of the NLRP3 inflammasome is a critical step. The NLRP3 inflammasome, as a representative inflammasome, leads to the activation of Caspase‐1 through its own proteolytic process. The substrates of activated Caspase‐1 not only cleave the precursor cytokines pro‐IL‐1β/18 into IL‐1β/18 but also cleave proteins of the GSDMs family into the pore‐forming active fragment GSDM‐NT, resulting in pyroptosis (Wang et al. 2025). This process releases inflammatory factors IL‐1β and IL‐18, thereby mediating the inflammatory response (Wang et al. 2025).

**FIGURE 8:**
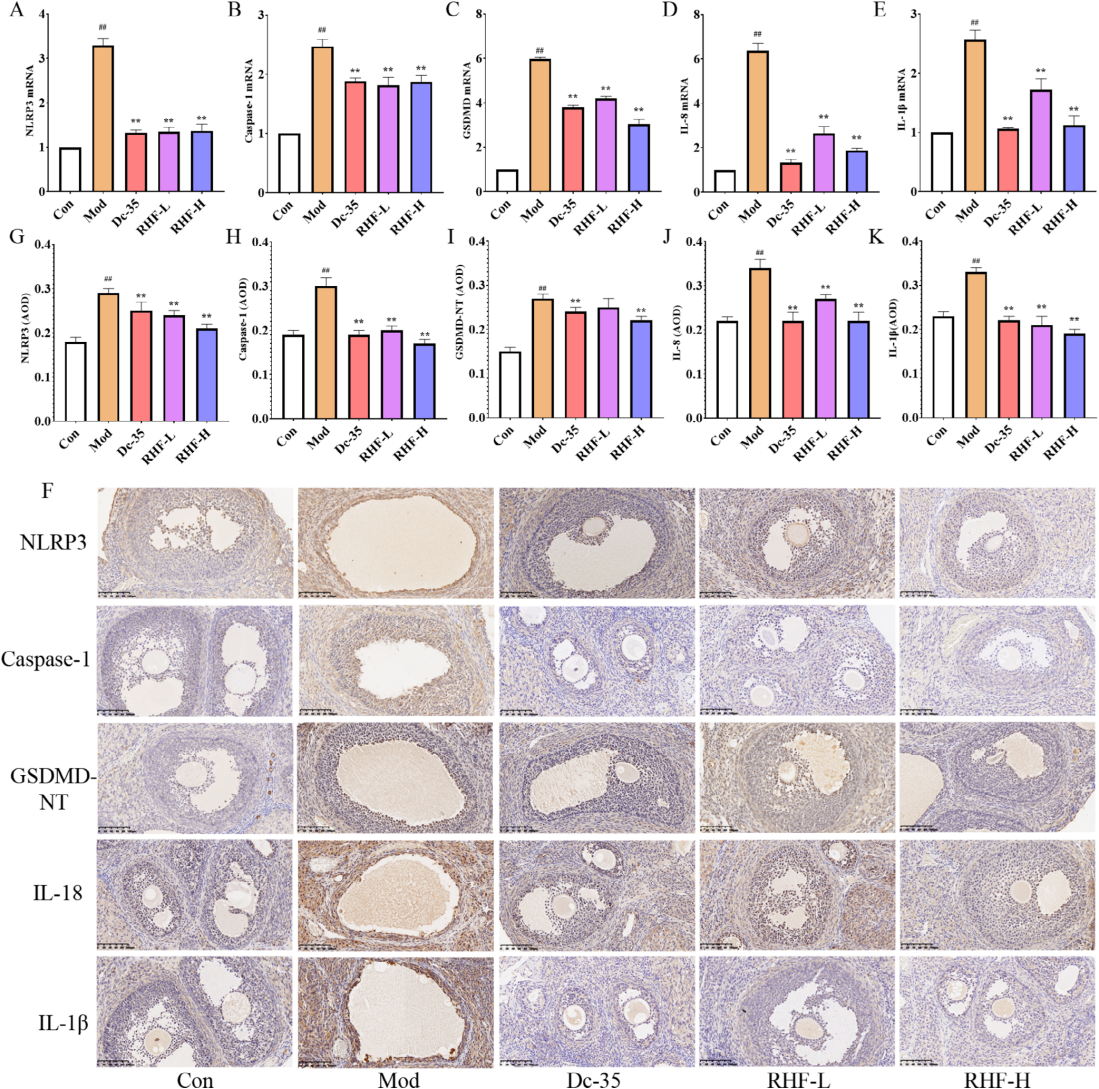
Effects of RHF on the expression of NLRP3, Caspase‐1, GSDMD‐NT, IL‐18, and IL‐1β in ovarian tissues of PCOS rats. (A) NLRP3 mRNA expression in the ovary. (B) Caspase‐1 mRNA expression in the ovary. (C) GSDMD mRNA expression in the ovary. (D) IL‐18 mRNA expression in the ovary. (E) IL‐1β mRNA expression in the ovary (*n* = 3). (F) Immunohistochemistry pictures. (G) NLRP3 protein expression in the ovary. (H) Caspase‐1 protein expression in the ovary. (I) GSDMD‐NT protein expression in the ovary. (J) IL‐18 protein expression in the ovary. (K) IL‐1β protein expression in the ovary. Data were shown as mean ± SD (*n* = 5). ^##^ *p* < 0.01 Con group versus the Mod group. *p < 0.05 versus the Mod group. **p < 0.01 versus the Mod group.

Our study revealed that compared with the Con group, the mRNA expression levels of NLRP3, Caspase‐1, GSDMD, IL‐18, and IL‐1β in ovarian tissues of the Mod group rats were significantly increased (*p* < 0.01). Compared with the Mod group, the mRNA expression levels of NLRP3, Caspase‐1, GSDMD, IL‐18, and IL‐1β in ovarian tissues of rats in each intervention group were significantly decreased (*p* < 0.01) (Figure 8A–E).

Subsequently, we further utilized IHC to observe the expression of related proteins in ovarian tissues. The results showed that compared with the Con group, the protein expression levels of NLRP3, Caspase‐1, GSDMD‐NT, IL‐18, and IL‐1β in the ovarian tissues of the Mod group rats were significantly increased (*p* < 0.01). Compared with the Mod group, the protein expression levels of NLRP3, Caspase‐1, GSDMD‐NT, IL‐18, and IL‐1β in the ovarian tissues of the Dc‐35 group and the RHF‐H group were significantly decreased (*p* < 0.01). In the RHF‐L group, the protein expression levels of NLRP3, Caspase‐1, IL‐18, and IL‐1β were significantly decreased (*p* < 0.01), while the change in GSDMD‐NT protein expression level was not statistically significant (*p* > 0.05) (Figure 8F–K). These results suggest that PCOS rats exhibit a local ovarian inflammatory response, and RHF can inhibit this local ovarian response in PCOS rats.

#### 3.5.3 Effect on the mRNA Levels of IL‐6 and TNF‐α in Ovarian Tissue of PCOS Rats

Chronic inflammation, as a significant factor influencing PCOS, is modulated by the body's androgen levels. It has been reported that a hyperandrogenic state stimulates the secretion of inflammatory factors such as IL‐6 and TNF‐α in the ovaries, leading to chronic inflammation in ovarian tissue. This disrupts the processes of follicular atresia and regression, ultimately resulting in ovulation disorders and the formation of cystic follicles (Rezvanfar et al. 2014). Furthermore, activation of the NLRP3 inflammasome not only promotes the release of inflammatory factors IL‐18 and IL‐1β but also increases the release of inflammatory factors TNF‐α and IL‐6, triggering an inflammatory cascade (Engin 2024).

**FIGURE 9:**
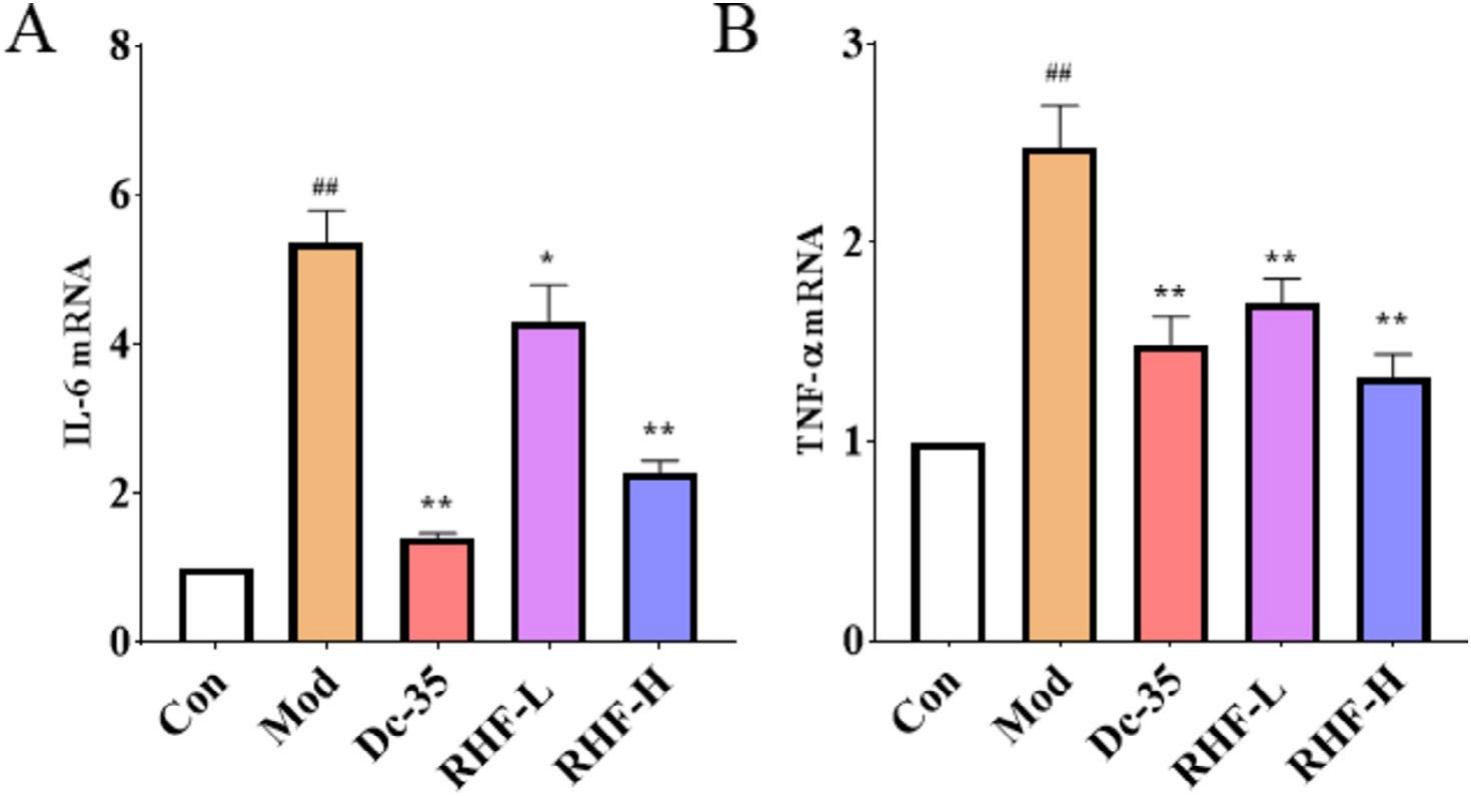
Effects of RHF on the expression of IL‐6 and TNF‐α in ovarian tissues of PCOS rats. (A) IL‐6 mRNA expression in the ovary. (B) TNF‐α mRNA expression in the ovary. Data were shown as mean ± SD (*n* = 3) ^##^ *p* < 0.01 Con group versus the Mod group. *p < 0.05 versus the Mod group. **p < 0.01 versus the Mod group.

Based on this, we further detected the mRNA expression levels of TNF‐α and IL‐6 in ovarian tissues. The results indicated that compared with the Con group, the mRNA expression levels of TNF‐α and IL‐6 in the ovarian tissues of rats in the Mod group were significantly increased (*p* < 0.01). Compared with the Mod group, the mRNA expression levels of TNF‐α and IL‐6 in the ovarian tissues of rats in both the Dc‐35 and RHF‐H groups were significantly decreased (*p* < 0.01). In the RHF‐L group, the mRNA expression level of IL‐6 in ovarian tissues was markedly decreased (*p* < 0.05), while the TNF‐α mRNA expression level was significantly decreased (*p* < 0.01) (Figure 9).

#### 3.5.4 Effect of RHF on the Serum Inflammatory Cytokine Levels (IL‐18, IL‐1β, IL‐6, and TNF‐α) in PCOS Rats

**FIGURE 10:**
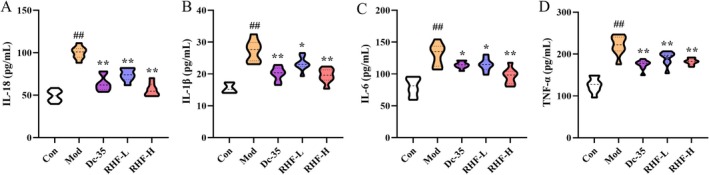
Effects of RHF on serum inflammatory cytokine levels (IL‐18, IL‐1β, IL‐6, and TNF‐α) in PCOS rats. (A) IL‐18 level in serum. (B) IL‐1β level in serum. (C) IL‐6 level in serum. (D) TNF‐α level in serum. Data were shown as mean ± SD (*n* = 8). ^##^ *p* < 0.01 Con group versus the Mod group. *p < 0.05 versus the Mod group. **p < 0.01 versus the Mod group.

Finally, serum levels of IL‐18, IL‐1β, IL‐6, and TNF‐α in PCOS rats were measured by ELISA. The results showed that the levels of these inflammatory cytokines were significantly elevated in the Mod group compared with the Con group (*p* < 0.01). Compared with the Mod group, serum levels of IL‐18, IL‐1β, and TNF‐α were significantly decreased in the Dc‐35 group (*p* < 0.01), while IL‐6 was markedly reduced (*p* < 0.05). In the RHF‐L group, IL‐18 and TNF‐α levels were significantly decreased (*p* < 0.01), whereas IL‐1β and IL‐6 showed a marked reduction (*p* < 0.05). Notably, in the RHF‐H group, all four cytokines—IL‐18, IL‐1β, IL‐6, and TNF‐α—were significantly decreased (*p* < 0.01) (Figure 10).

## 4 Discussion

PCOS is a complex endocrine disorder characterized by not only impacting female reproductive health, causing ovulatory dysfunction and even infertility, but also increasing the risk of various diseases such as metabolic syndrome, type 2 diabetes, cardiovascular diseases, and mood disorders (Helvaci and Yildiz 2025). Due to its intricate pathogenesis and diverse clinical manifestations, there are currently no specific medications or curative treatments available for PCOS. The primary therapeutic goals focus on the restoration of regular menstrual cycles, balancing hormone secretion, improving metabolic parameters, and suppressing inflammatory responses.

The HPO axis, a key regulatory system in the female reproductive system, represents a critical pathophysiological mechanism in PCOS. It is closely associated with clinical manifestations in PCOS patients, such as menstrual disorders, hormonal imbalances, and ovarian pathological changes (Zhang, Jiang, et al. 2025). Among these, menstrual irregularity is one of the most common symptoms in PCOS patients, manifesting as irregular menstrual cycles, intermittent menstruation, or prolonged amenorrhea. This is primarily due to ovarian dysfunction, which prevents normal follicular development and ovulation, thereby disrupting corpus luteum formation and the secretion of estrogen and progesterone, consequently leading to menstrual abnormalities (Tian and Chen 2025). Disordered sex hormone secretion is another key clinical feature of PCOS. Patients typically exhibit elevated T levels and decreased E_2_ levels (Bushell and Crespi 2024). Elevated T levels provide feedback to the hypothalamus, increasing the frequency of GnRH release. This increased GnRH pulsatility leads to an imbalance in pituitary gland secretion of FSH and LH. Proper secretion of FSH and LH is crucial for normal oocyte development. FSH levels are inversely correlated with ovarian reserve function, while LH promotes androgen secretion and their conversion to estrogen, facilitating follicular development and maturation. However, elevated LH can also stimulate theca cells to excessively secrete T, thereby exacerbating PCOS (Hu et al. 2025; Zhang, Huang, et al. 2025). E_2_, a crucial estrogen in female fertility, is closely associated with impaired follicular development and ovulation abnormalities when its levels are decreased. Furthermore, reduced E_2_ levels are also linked to endometrial hypoplasia and diminished endometrial receptivity (Yun et al. 2024). It is noteworthy that the ovary, as the primary target organ in PCOS pathology, undergoes not only microscopic pathological changes under the influence of various factors but also often exhibits an increase in both size and weight (Peng et al. 2021). Abnormal apoptosis affects follicular development and is involved in the pathogenesis of PCOS. Among the key regulators, Bcl‐2 family proteins are crucial for maintaining mitochondrial integrity by regulating the balance between pro‐apoptotic and anti‐apoptotic signals. Specifically, Bax can increase mitochondrial membrane permeability and alter the transmembrane potential, leading to the release of cytochrome c from the mitochondria into the cytoplasm. This triggers a caspase cascade and ultimately activates Caspase‐3, inducing cell apoptosis (Zhang et al. 2019). In contrast, Bcl‐2 protein exerts anti‐apoptotic effects by inhibiting the activation of a series of signals initiated by pro‐apoptotic proteins (Vardiyan et al. 2020). Therefore, the expression levels of Bcl‐2, Bax, and Caspase‐3 can, to some extent, reflect the recovery status of the ovary. Consequently, restoring regular menstrual cycles, correcting hormonal imbalances, inhibiting ovarian hyperplasia, and alleviating ovarian pathological changes are of positive significance for the treatment of PCOS. In this study, we found that RHF restored the disrupted estrous cycle in PCOS rats, increased E_2_ and LH levels, decreased GnRH, FSH, and T levels, reduced ovarian weight, ovarian index, ovarian length and width, ovarian area and volume, increased the number of corpora lutea in ovarian tissues, and decreased the number of cystic and atretic follicles, thereby alleviating pathological changes. These results indicate that RHF can improve the endocrine function of the HPO axis in PCOS rats. Furthermore, we observed that RHF increased SHBG levels. As a key protein regulating sex hormone levels in the body, SHBG binds to sex hormones such as T and E_2_, thereby influencing their bioavailability, metabolic rate, and subsequent biological effects (Sun et al. 2024). This suggests that RHF may correct sex hormone imbalances in PCOS through two potential pathways: improving HPO axis function and modulating SHBG secretion. However, the specific mechanisms of action require further investigation.

Kelly et al. (2001) proposed the inflammatory theory and were the first to introduce it into the pathogenesis of PCOS. Routine blood tests are the most common examination in clinical disease diagnosis, widely used in clinical practice, and serve as a common indicator reflecting systemic inflammatory responses (Zhang et al. 2024). Recent studies have confirmed that peripheral blood cell inflammatory markers (WBC, PLT, RDW, NLR, MLR, and PLR) are abnormally expressed in PCOS patients, thereby demonstrating the existence of a systemic inflammatory response in PCOS (Yang et al. 2023). Elevated levels of inflammatory cytokines such as TNF‐α, IL‐1β, IL‐6, and IL‐18 in the body can promote an increase in androgen levels, exacerbate inflammatory responses, inhibit oocyte maturation, and consequently affect metabolic and reproductive functions (Vaziri Esfarjani et al. 2025). Among various inflammatory factors, IL‐1β can promote the expression of key enzymes for androgen synthesis in ovarian granulosa cells and theca cells, thereby accelerating androgen production and secretion. Elevated IL‐1β levels further exacerbate local ovarian inflammation, while high androgen levels combined with local ovarian inflammation lead to follicular developmental arrest, anovulation or ovulatory dysfunction, ultimately impairing follicular development and ovulation processes (Zhang, Huang, et al. 2025). IL‐18 is a potent pro‐inflammatory cytokine capable of initiating and sustaining a chronic low‐grade inflammatory state in PCOS patients. This persistent inflammatory response can impair ovarian function, leading to ovulatory dysfunction and metabolic disturbances, thereby contributing to the formation of typical PCOS characteristics (Zhang, Wang, et al. 2025). It has been reported that serum IL‐18 levels are significantly higher in PCOS patients compared to healthy controls, and these levels show an upward trend with increasing disease severity (Ma et al. 2024). Among various inflammatory signaling pathways, the NLRP3 inflammasome signaling pathway—composed of NLRP3, ASC, and Caspase‐1—is a key pathway in the innate immune system responsible for inflammatory responses. It primarily regulates the levels of IL‐1β and IL‐18 in the body (Siddiqua et al. 2025). Upon stimulation by various factors, NLRP3 is activated and assembles with ASC to form the inflammasome. Subsequently, ASC recruits and activates Caspase‐1, which cleaves pro‐IL‐1β and pro‐IL‐18 to generate mature IL‐1β and IL‐18. This process further promotes the release of TNF‐α and IL‐6, thereby initiating inflammatory responses and contributing to the pathological progression of PCOS (Samadi Nasab et al. 2025). Therefore, improving hematological parameters, balancing blood cell ratios, and inhibiting the activation of the NLRP3 inflammasome signaling pathway hold significant therapeutic implications for the treatment of PCOS. We found that administration of RHF significantly lowered peripheral blood indices (WBC, PLT, RDW, NLR, MLR, PLR) and serum levels of IL‐18, IL‐1β, IL‐6, and TNF‐α. Moreover, RHF suppressed the expression of NLRP3, Caspase‐1, IL‐18, and IL‐1β at both the protein and mRNA levels in the ovaries, while also inhibiting ovarian TNF‐α and IL‐6 mRNA expression. This suggests that RHF effectively mitigates systemic inflammation and inhibits local ovarian inflammatory responses in PCOS rats. In conclusion, our study suggests that RHF exerts a protective effect in PCOS rats by improving HPO axis function and inhibiting inflammatory responses.

However, this study has several limitations that warrant further exploration and refinement in the future. At the mechanistic level, the specific pathways through which RHF exerts its effects, as well as the key active components within its multi‐constituent mixture, remain to be fully elucidated. Methodologically, serum hormone sampling was not standardized to a specific stage of the estrous cycle, which may affect the accurate interpretation of indicators such as E_2_ and T. Regarding the measurement of the main components of RHF, although HPLC analysis qualitatively identified the presence of key components, the precise concentrations (mg/g) were not determined. This omission may affect the reproducibility of the extract's chemical profile and hinder the establishment of a direct dose–response relationship for specific monomers. Future investigations should prioritize the standardization of RHF extracts through comprehensive quantitative assays to facilitate clinical translation. These issues, pertaining to both mechanism and experimental technique, will be the focus of our subsequent research. Moreover, this study primarily focuses on the regulation of the HPO axis and the NLRP3 inflammasome signaling pathway by RHF. It is known that oxidative stress plays a critical role in the pathogenesis of PCOS (Zeber‐Lubecka et al. 2023) and the activation of the NLRP3 inflammasome (Wang et al. 2023), while flavonoids generally possess antioxidant activity. Therefore, whether RHF indirectly inhibits the NLRP3 inflammasome by alleviating oxidative stress, thereby improving endocrine function, is an interesting hypothesis postulated by this study that awaits direct verification in future research.

## 5 Conclusion

RHF can improve the imbalance of sex hormone secretion, restore the estrous cycle, inhibit ovarian hyperplasia and pathological changes, and alleviate systemic and local ovarian inflammatory responses in PCOS rats. These effects are accompanied by improvements in HPO axis function and a reduction in the activation level of the NLRP3 inflammasome signaling pathway, suggesting that both the HPO axis and the NLRP3 inflammasome signaling pathway may jointly participate in the above processes. This also provides an important direction for further validation of its specific mechanisms through pathway inhibition experiments and other in‐depth studies.

## Author Contributions

**Baosong Liu:** writing – original draft, methodology, formal analysis, conceptualization, data curation. **Chuan Rao:** writing – review and editing. **Lanyue Cao:** writing – review and editing. **Mengfan Peng:** writing – original draft, methodology, funding acquisition, formal analysis, conceptualization. **Lei Yang:** funding acquisition, project administration, data curation. **Caixia Li:** methodology, writing – review and editing, project administration. **Yingying Sun:** methodology, formal analysis. **Hao Chen:** funding acquisition, project administration, methodology.

## Funding

This work was supported by Henan Science and Technology Research (No. 242102310578, 262102310533, 262102310521, 252102310525), Young Scientists Fund Project of the National Natural Science Foundation of China (No. 82505164), Natural Science Foundation Project of Henan Province (No. 252300423830) and the Major Science and Technology Projects in Zhumadian City (grant no. [2024]337).

## Ethics Statement

All procedures involving rats were approved by the Ethics Committee of Huanghuai University and the experimental animal ethics Batch number is 2023102303.

## Conflicts of Interest

The authors declare no conflicts of interest.

## Data Availability

Data will be made available on request.

## References

[fsn372004-bib-0001] Bushell, A. , and B. J. Crespi . 2024. “The Evolutionary Basis of Elevated Testosterone in Women With Polycystic Ovary Syndrome: An Overview of Systematic Reviews of the Evidence.” Frontiers in Reproductive Health 6: 1475132. 10.3389/frph.2024.1475132. 39403367 PMC11471738

[fsn372004-bib-0002] Cao, K. X. 2024. “Advances in Molecular Genetics and Breeding of Cattle, Sheep, and Goats.” Animals 16, no. 8: 1130. 10.3390/ani16081130. PMC13113644 42071900

[fsn372004-bib-0003] Danduga, R. C. S. R. , A. S. Kurapati , R. A. Shaik , P. K. Kola , S. K. Konidala , and H. B. Varada . 2024. “Synergistic Amelioration of Letrozole‐Induced Polycystic Ovary Syndrome in Rats: A Therapeutic Approach With Apple Cider Vinegar and Metformin Combination.” Reproductive Sciences 31, no. 9: 2861–2876. 10.1007/s43032-024-01545-4. 38777948

[fsn372004-bib-0004] Deng, H. , Y. Chen , J. Xing , N. Zhang , and L. Xu . 2024. “Systematic Low‐Grade Chronic Inflammation and Intrinsic Mechanisms in Polycystic Ovary Syndrome.” Frontiers in Immunology 15: 1470283. 10.3389/fimmu.2024.1470283. 39749338 PMC11693511

[fsn372004-bib-0005] Dong, S. Q. , Y. Q. Tang , H. T. Xu , X. L. Kong , B. Fu , and Q. Tong . 2025. “Effect of Qigong Pill on the AdipoR/AMPK Signaling Pathway in Polycystic Ovary Syndrome‐Insulin Resistant Rats.” Chinese Traditional Patent Medicine 47, no. 2: 584–589. 10.3969/j.issn.1001-1528.2025.02.037.

[fsn372004-bib-0006] Dou, J. , Y. Wu , R. Hu , et al. 2024. “Quinoa Ameliorates Polycystic Ovary Syndrome via Regulating Gut Microbiota Through PI3K/AKT/mTOR Pathway and Autophagy.” Nutrition & Metabolism 21, no. 1: 80. 10.1186/s12986-024-00855-3. 39394588 PMC11468221

[fsn372004-bib-0007] Engin, A. 2024. “Reappraisal of Adipose Tissue Inflammation in Obesity.” Advances in Experimental Medicine and Biology 1460: 297–327. 10.1007/978-3-031-63657-8_10. 39287856

[fsn372004-bib-0008] He, B. , L. Dai , L. Jin , et al. 2023. “Bioactive Components, Pharmacological Effects, and Drug Development of Traditional Herbal Medicine *Rubus chingii* Hu (Fu‐Pen‐Zi).” Frontiers in Nutrition 9: 1052504. 36698464 10.3389/fnut.2022.1052504 PMC9868258

[fsn372004-bib-0009] Helvaci, N. , and B. O. Yildiz . 2025. “Polycystic Ovary Syndrome as a Metabolic Disease.” Nature Reviews. Endocrinology 21, no. 4: 230–244. 10.1038/s41574-024-01057-w. 39609634

[fsn372004-bib-0010] Hen, M. S. , X. Y. Jia , D. J. Hou , et al. 2025. “Fabrication of Rubus Chingii Hu Ellagitannins‐Loaded W/O and O/W Emulsion Gels: Structure, Stability, in Vitro Digestion and in Vivo Metabolism.” International Journal of Biological Macromolecules 295: 139656. 10.1016/j.ijbiomac.2025.139656. 39793811

[fsn372004-bib-0011] Hu, R. , Y. Geng , Y. Huang , et al. 2024. “Jiawei Buzhong Yiqi Decoction Attenuates Polycystic Ovary Syndrome Through Regulating Kisspeptin‐GPR54‐AKT‐ SHBG System.” Phytomedicine: International Journal of Phytotherapy and Phytopharmacology 133: 155931. 10.1016/j.phymed.2024.155931. 39116604

[fsn372004-bib-0012] Hu, R. , Y. Huang , Z. Liu , et al. 2025. “Characteristics of Polycystic Ovary Syndrome Rat Models Induced by Letrozole, Testosterone Propionate and High‐Fat Diets.” Reproductive Biomedicine Online 50, no. 1: 104296. 10.1016/j.rbmo.2024.104296. 39626468

[fsn372004-bib-0013] Jafar, N. K. A. , M. Fan , L. J. Moran , D. R. Mansfield , and C. J. Bennett . 2025. “Sex Hormones, Sex Hormone‐Binding Globulin and Sleep Problems in Females With Polycystic Ovary Syndrome: A Systematic Review and Meta‐Analysis.” Clinical Endocrinology 102, no. 6: 708–720. 10.1111/cen.15219. 39996383 PMC12046544

[fsn372004-bib-0014] Jia, R. X. , L. Lv , H. Y. Liu , et al. 2025. “Diagnostic Value of Blood Cell Count‐Related Biomarkers in predicting28‐Day Mortality in Acute‐On‐Chronic Hepatitis b Liver Failure.” Journal of Shandong University (Medical Edition) 63, no. 6: 89–99. 10.6040/j.issn.1671-7554.2024.1354.

[fsn372004-bib-0015] Kelly, C. C. , H. Lyall , J. R. Petrie , G. W. Gould , J. M. Connell , and N. Sattar . 2001. “Low Grade Chronic Inflammation in Women With Polycystic Ovarian Syndrome.” Journal of Clinical Endocrinology and Metabolism 86, no. 6: 2453–2455. 10.1210/jcem.86.6.7580. 11397838

[fsn372004-bib-0016] Li, H. Z. 2021. “Role of Acupoint Catgut Embedding and Rubus Chingiiin Improving PCOS‐Related Insulin Resistance Through the TXNIP‐NLRP3 Pathway.” Qinghai University. 10.27740/d.cnki.gqhdx.2021.000088.

[fsn372004-bib-0017] Li, S. W. , Y. Z. Liao , L. F. Wang , et al. 2025. “Quality Evaluation of Rubi Fructus Based on UPLC‐Q‐TOF‐MS/MS,HPLC Fingerprints and Content Determination.” Chinese Traditional Patent Medicine 47, no. 4: 1077–1084. 10.3969/j.issn.1001-1528.2025.04.002.

[fsn372004-bib-0018] Ma, L. X. , Q. Wang , Y. K. Wang , and Z. M. Lu . 2024. “Association of Serum IL‐18, CTRP3, and HSP70 Levels With Disease Severity and Prognosis in PCOS.” Journal of Medical Theory and Practice 37, no. 22: 3900–3903. 10.19381/j.issn.1001-7585.2024.22.042.

[fsn372004-bib-0019] Magdy, N. , N. F. Abdelkader , H. F. Zaki , and A. S. Kamel . 2024. “Potential Exacerbation of Polycystic Ovary Syndrome by Saccharin Sodium via Taste Receptors in a Letrozole Rat Model.” Food and Chemical Toxicology: An International Journal Published for the British Industrial Biological Research Association 191: 114874. 10.1016/j.fct.2024.114874. 39032681

[fsn372004-bib-0020] Peng, M. F. , S. Tian , Y. G. Song , et al. 2021. “Effects of Total Flavonoids From *Eucommia ulmoides* Oliv. Leaves on Polycystic Ovary Syndrome With Insulin Resistance Model Rats Induced by Letrozole Combined With a High‐Fat Diet.” Journal of Ethnopharmacology 273: 113947. 10.1016/j.jep.2021.113947. 33617969

[fsn372004-bib-0021] Rezvanfar, M. A. , H. A. Shojaei Saadi , M. Gooshe , A. H. Abdolghaffari , M. Baeeri , and M. Abdollahi . 2014. “Ovarian Aging‐Like Phenotype in the Hyperandrogenism ‐Induced Murine Model of Polycystic Ovary.” Oxidative Medicine and Cellular Longevity 2014: 948951. 10.1155/2014/948951. 24693338 PMC3945218

[fsn372004-bib-0022] Samadi Nasab, F. , H. Babei , M. Nayebzadeh , et al. 2025. “NLRP3 Inflammasome Activation in PCOS: A Novel Target for Managing Insulin Resistance and Metabolic Dysregulation.” Tissue & Cell 97: 103097. 10.1016/j.tice.2025.103097. 40857871

[fsn372004-bib-0023] Siddiqua, A. , A. Malik , and U. Iqbal . 2025. “Modulating Endoplasmic Reticulum Stress and NLRP3 Inflammasome in Polycystic Ovary Syndrome: A Review of Natural and Synthetic Compounds.” Inflammopharmacology 33, no. 8: 4519–4533. 10.1007/s10787-025-01875-y. 40759851

[fsn372004-bib-0024] Simon, S. L. , P. Phimphasone‐Brady , K. M. McKenney , et al. 2024. “Comprehensive Transition of Care for Polycystic Ovary Syndrome From Adolescence to Adulthood. The Lancet.” Child & Adolescent Health 8, no. 6: 443–455. 10.1016/S2352-4642(24)00019-1. 38552655 PMC11837223

[fsn372004-bib-0025] Su, P. , C. Chen , and Y. Sun . 2025. “Physiopathology of Polycystic Ovary Syndrome in Endocrinology, Metabolism and Inflammation.” Journal of Ovarian Research 18, no. 1: 34. 10.1186/s13048-025-01621-6. 39980043 PMC11841159

[fsn372004-bib-0026] Sun, S. , Y. Liu , L. Li , et al. 2024. “Unveiling the Shared Genetic Architecture Between Testosterone and Polycystic Ovary Syndrome.” Scientific Reports 14, no. 1: 23931. 10.1038/s41598-024-75816-0. 39397165 PMC11471787

[fsn372004-bib-0027] Tian, Y. , and F. Q. Chen . 2025. “Levels of Serum Sex Hormones, Glycolipid Metabolic Markers, and Immune Markers in Infertile Women With Polycystic Ovary Syndrome.” Chinese Journal of Woman and Child Health 40, no. 17: 3211–3215. 10.19829/j.zgfybj.issn.1001-4411.2025.17.026.

[fsn372004-bib-0028] Valera, H. , A. Chen , and K. J. Grive . 2025. “The Hypothalamic‐Pituitary‐Ovarian Axis, Ovarian Disorders, and Brain Aging.” Endocrinology 166: bqaf137. 10.1210/endocr/bqaf137. 40884186 PMC12448947

[fsn372004-bib-0029] Vardiyan, R. , D. Ezati , M. Anvari , N. Ghasemi , and A. Talebi . 2020. “Effect of L‐Carnitine on the Expression of the Apoptotic Genes Bcl‐2 and Bax.” Clinical and Experimental Reproductive Medicine 47, no. 3: 155–160. 10.5653/cerm.2019.03440. 32911587 PMC7482949

[fsn372004-bib-0030] Vaziri Esfarjani, F. , P. Dorfeshan , A. Mansouri , et al. 2025. “Association Between Dietary Inflammatory Index, Empirical Dietary Inflammatory Patterns, Dietary and Lifestyle Inflammation Scores, and Polycystic Ovary Syndrome: A Case‐Control Study.” BMC Endocrine Disorders 25, no. 1: 202. 10.1186/s12902-025-02022-y. 40903727 PMC12409934

[fsn372004-bib-0031] Wang, C. , X. Q. Zhou , X. Liu , and Z. Y. Yin . 2025. “Apelin‐13 Inhibits RANKL‐Induced Macrophage Differentiation Into Oste Oclasts via the Nrf2‐ Pyroptosis Pathway.” Chinese Journal of Osteoporosis 31, no. 3: 343–348+408. 10.3969/j.issn.1006-7108.2025.03.005.

[fsn372004-bib-0032] Wang, Y. , J. Yang , Y. Wang , et al. 2023. “Upregulation of TXNIP Contributes to Granulosa Cell Dysfunction in Polycystic Ovary Syndrome via Activation of the NLRP3 Inflammasome.” Molecular and Cellular Endocrinology 561: 111824. 10.1016/j.mce.2022.111824. 36450326

[fsn372004-bib-0033] Wu, Y. X. , X. Y. Yang , B. S. Han , et al. 2022. “Naringenin Regulates Gut Microbiota and SIRT1/ PGC‐1ɑ Signaling Pathway in Rats With Letrozole‐Induced Polycystic Ovary Syndrome.” Biomedicine & Pharmacotherapy = Biomedecine & Pharmacotherapie 153: 113286. 10.1016/j.biopha.2022.113286. 35724506

[fsn372004-bib-0034] Yang, H. , S. Y. Lee , S. R. Lee , et al. 2018. “Therapeutic Effect of Ecklonia Cava Extract in Letrozole‐Induced Polycystic Ovary Syndrome Rats.” Frontiers in Pharmacology 9: 1325. 10.3389/fphar.2018.01325. 30524282 PMC6262357

[fsn372004-bib-0035] Yang, X. H. , Y. J. Liu , H. Q. Zhang , and Y. Y. Peng . 2023. “Study on the Relationship Between Peripheral Blood Cell Inflammatory Markers NLR,PLR,MLR,dNLR,SII and PCOS.” International Journal of Laboratory Medicine 44, no. 22: 2768–2772 CNKI:SUN:GWSQ.0.2023–22‐015.

[fsn372004-bib-0036] Yun, R. Y. , W. Zhu , and M. Xue . 2024. “Effect of Estradiol Valerate Combined With Cyproterone Acetate and Ethinylestradiol Tablets on Polycystic Ovary Syndrome and Its Influence on Sex Hormone Levels.” Doctor 9, no. 15: 4–7.

[fsn372004-bib-0037] Zeber‐Lubecka, N. , M. Ciebiera , and E. E. Hennig . 2023. “Polycystic Ovary Syndrome and Oxidative Stress‐From Bench to Bedside.” International Journal of Molecular Sciences 24, no. 18: 14126. 10.3390/ijms241814126. 37762427 PMC10531631

[fsn372004-bib-0038] Zhang, H. X. , L. P. Wang , Y. R. Cao , and M. M. Feng . 2025. “Improvement of Ovarian Function by Ginkgolides in PCOS Rats via the TLR4/MyD88/NF‐κB Pathway.” Pharmacology and Clinics of Chinese Materia Medica 42, no. 4: 51–58. 10.13412/j.cnki.zyyl.20250929.003.

[fsn372004-bib-0039] Zhang, J. , H. Huang , M. Xiao , et al. 2025. “Erchen Decoction Ameliorates the Rat Model of Polycystic Ovary Syndrome by Regulating the Steroid Biosynthesis Pathway.” Phytomedicine: International Journal of Phytotherapy and Phytopharmacology 143: 156852. 10.1016/j.phymed.2025.156852. 40446578

[fsn372004-bib-0040] Zhang, S. , N. Jiang , G. Liu , et al. 2025. “New Perspectives on Polycystic Ovary Syndrome: Hypothalamic‐Sympathetic‐Adipose Tissue Interaction.” Journal of Ovarian Research 18, no. 1: 145. 10.1186/s13048-025-01724-0. 40615863 PMC12232167

[fsn372004-bib-0041] Zhang, X. J. , M. Chen , Y. H. Feng , X. L. Du , D. J. Ji , and H. M. Ma . 2023. “Improving Effect of Jianpi Yishen Huazhuo Recipe on Ovarian Function in Rats With Polycystic Ovary Syndrome Based on Bax/Bcl‐2/Caspase‐3 Pathway.” Chinese Journal of Modern Applied Pharmacy 40, no. 20: 2891–2896. 10.13748/j.cnki.issn1007-7693.20223449.

[fsn372004-bib-0042] Zhang, Y. , T. Li , Q. Chen , M. Shen , X. Fu , and C. Liu . 2024. “The Relationship Between Complete Blood Cell Count‐Derived Inflammatory Biomarkers and Erectile Dysfunction in the United States.” Scientific Reports 14, no. 1: 32014. 10.1038/s41598-024-83733-5. 39738513 PMC11685723

[fsn372004-bib-0043] Zhang, Y. , X. Yang , X. Ge , and F. Zhang . 2019. “Puerarin Attenuates Neurological Deficits via Bcl‐2/Bax/Cleaved Caspase‐3 and Sirt3/SOD2 Apoptotic Pathways in Subarachnoid Hemorrhage Mice.” Biomedicine & Pharmacotherapy = Biomedecine & Pharmacotherapie 109: 726–733. 10.1016/j.biopha.2018.10.161. 30551525

[fsn372004-bib-0044] Zuchelo, L. T. S. , M. S. Alves , E. C. Baracat , I. C. E. Sorpreso , and J. M. Soares . 2024. “Menstrual Pattern in Polycystic Ovary Syndrome and Hypothalamic‐ Pituitary‐Ovarian Axis Immaturity in Adolescents: A Systematic Review and Meta‐Analysis.” Gynecological Endocrinology: The Official Journal of the International Society of Gynecological Endocrinology 40, no. 1: 2360077. 10.1080/09513590.2024.2360077. 38818646

